# Circulating Soluble Urokinase Plasminogen Activator Receptor and C-Reactive Protein Concentrations in Adults with High-Functioning Autism

**DOI:** 10.3390/brainsci16101100

**Published:** 2026-10-12

**Authors:** Ayşe Kurtulmuş, Beyzanur Ereğli, Betül Nas Dönmez, Aynur Görmez

**Affiliations:** 1 Department of Psychiatry, Faculty of Medicine, Istanbul Medeniyet University, Eğitim Mah. Fahrettin Kerim Gökay Caddesi, Kadıköy, Istanbul 34722, Turkey; 2 Department of Genetics, Aziz Sancar Institute of Experimental Medicine, Istanbul University, Istanbul 34093, Turkey

**Keywords:** autism spectrum disorder, high-functioning autism, suPAR, CRP

## Abstract

**Background:** Immune dysregulation has been implicated in the pathophysiology of autism spectrum disorder (ASD). Soluble urokinase plasminogen activator receptor (suPAR) is a stable biomarker of chronic immune activation that has not previously been investigated in ASD. We compared circulating suPAR and C-reactive protein (CRP) concentrations in adults with high-functioning ASD and healthy controls. **Methods:** In this cross-sectional case–control study, 38 adults with high-functioning ASD and 37 healthy controls were included. Plasma suPAR and CRP concentrations were measured using enzyme-linked immunosorbent assays. Group differences were examined using hierarchical multiple linear regression analyses adjusting for age, sex, body mass index (BMI), and current smoking status. The Autism Spectrum Quotient (AQ) was administered to all participants, and associations between inflammatory biomarkers and autistic traits were assessed using Spearman’s rank correlation analyses. Sensitivity analyses were performed after excluding participants with CRP concentrations > 10 mg/L to minimise the potential influence of acute inflammatory conditions. **Results:** In unadjusted analyses, CRP concentrations were significantly higher in the ASD group (*p* = 0.006), whereas suPAR concentrations did not differ (*p* = 0.163). After adjustment for age, sex, BMI, and smoking status, the association between ASD and CRP was no longer significant (*p* = 0.181), with BMI emerging as the strongest independent predictor of CRP (*p* < 0.001). No association was observed between ASD and circulating suPAR concentrations in either unadjusted or adjusted analyses (*p* = 0.612). CRP, but not suPAR, was positively correlated with AQ total scores (*p* = 0.026). Sensitivity analyses excluding participants with high CRP concentrations yielded findings consistent with the primary analyses. **Conclusions:** Adults with high-functioning ASD showed no evidence of elevated circulating suPAR concentrations. The attenuation of CRP group differences after adjustment for BMI and smoking status highlights the importance of accounting for metabolic factors when interpreting peripheral inflammatory biomarkers in ASD. These findings do not support elevated suPAR levels in adults with high-functioning ASD.

## 1. Introduction

Autism spectrum disorder (ASD) is a heterogeneous neurodevelopmental illness characterised by persistent difficulties in social communication and social interaction, restricted and repetitive patterns of behaviour or interests, and sensory processing abnormalities [1]. Recent epidemiological estimates indicate that ASD affects approximately 1 in 36 individuals, making it one of the most prevalent neurodevelopmental disorders worldwide [2]. Despite substantial advances in genetic, neurodevelopmental, and neurobiological research, the biological mechanisms underlying ASD remain incompletely understood. Consequently, considerable effort has been devoted to identifying reliable biomarkers that may improve our understanding of ASD pathophysiology. Given both the increasing prevalence of ASD and its considerable personal and societal burden, elucidating the biological pathways involved in its pathophysiology has become a major research priority.

Increasing evidence suggests that immune dysregulation and inflammatory processes may be important in the pathophysiology of ASD. Peripheral and central immune activation abnormalities have been reported in prior studies, including elevated circulating inflammatory cytokines, alterations in leukocyte profiles, chronic low-grade systemic inflammation, and microglial activation [3,4,5,6]. Persistent immune activation has been proposed to influence neurodevelopment, synaptic plasticity, and neuronal connectivity, thereby potentially contributing to the pathophysiology of ASD [6,7,8,9]. Nevertheless, findings regarding peripheral inflammatory biomarkers in ASD remain inconsistent. Although this inconsistency is likely multifactorial, an important challenge in psychiatric biomarker research is that circulating inflammatory markers are influenced by numerous biological and environmental factors, including acute infections, sleep–wake disturbances, medication use, lifestyle factors, metabolic status and medical comorbidities. Consequently, distinguishing disease-related inflammatory alterations from those attributable to these confounding factors remains difficult, highlighting the need for biomarkers that more reliably reflect persistent immune activation.

In addition to these methodological challenges, the marked biological and clinical heterogeneity of ASD represents another major obstacle to biomarker research. Individuals with ASD exhibit substantial variability in symptom severity, intellectual functioning, language development, adaptive abilities, and psychiatric and medical comorbidities. Increasing evidence suggests that these phenotypic differences may reflect distinct underlying biological and etiopathogenetic mechanisms rather than a single uniform disease process [10,11,12,13]. Therefore, biomarker studies including heterogeneous ASD populations may obscure biologically meaningful subgroup-specific alterations. High-functioning ASD represents a relatively homogeneous clinical phenotype characterised by preserved intellectual functioning despite persistent impairments in social communication and restricted or repetitive behaviours/interests. Accordingly, focusing on high-functioning ASD may provide a more appropriate framework for investigating phenotype-specific biological mechanisms and identifying clinically relevant biomarkers. Nevertheless, inflammatory alterations in this clinically important subgroup remain poorly characterised, particularly in adults. To date, only a few studies have specifically investigated inflammatory alterations in individuals with high-functioning ASD or related phenotypes. Suzuki et al. (2011) reported increased circulating levels of several cytokines, including IL-1β, IL-1RA, IL-5, IL-8, IL-12p70, IL-13, IL-17, and GRO-α, in a small sample of non-medicated adolescent males with high-functioning ASD [14], while a subsequent study in adults with high-functioning ASD found increased TNF-α expression in differentiated M1 macrophages but not in circulating monocytes [15]. In another study, comprehensive profiling of serum biomarkers in adults with Asperger syndrome revealed sex-specific inflammatory signatures, with increased cytokines and other inflammatory molecules observed only in males [16]. Collectively, these findings suggest that inflammatory alterations may be present in high-functioning ASD but also highlight the limited evidence base and the potential influence of sex on inflammatory biomarker profiles.

Soluble urokinase plasminogen activator receptor (suPAR) has gained increasing attention as a stable indicator of chronic immune activation. suPAR is generated through proteolytic cleavage of the membrane-bound urokinase plasminogen activator receptor (uPAR) during immune activation and reflects persistent activation of the innate immune system. Compared with many conventional inflammatory biomarkers, circulating suPAR concentrations appear to be relatively stable and less susceptible to short-term physiological fluctuations associated with acute inflammatory responses, medication use, circadian variation, and lifestyle-related factors [17]. These characteristics have led to increasing interest in suPAR as a biomarker of chronic low-grade inflammation across a wide range of medical disorders [18,19]. Elevated circulating suPAR levels have also been reported in schizophrenia and mood disorders, suggesting that chronic immune activation could represent a common biological mechanism across psychiatric diseases [20]. Given its distinct biological characteristics, evaluating suPAR may enhance our understanding of chronic immune activation in ASD and provide complementary information to conventional inflammatory biomarkers.

Despite growing evidence implicating inflammatory mechanisms in ASD, studies investigating inflammatory biomarkers in adults with high-functioning ASD remain scarce, and the potential role of suPAR in this population has not yet been explored. Therefore, the primary aim of the present study was to compare circulating suPAR concentrations between adults with high-functioning ASD and healthy controls, while simultaneously assessing CRP as a conventional inflammatory biomarker to provide complementary information on inflammatory status. By focusing on a clinically more homogeneous subgroup, we sought to minimise biological heterogeneity and improve the detection of phenotype-specific inflammatory alterations. As a secondary aim, we examined the associations between inflammatory biomarkers and autistic traits. We hypothesized that adults with high-functioning ASD would exhibit increased circulating inflammatory biomarker concentrations compared with healthy controls and that these biomarkers would be associated with autistic traits.

## 2. Materials and Methods

### 2.1. Participants

This case–control study included 38 adults with high-functioning autism spectrum disorder (ASD) and 37 healthy controls. Participants with ASD were consecutively recruited according to predefined eligibility criteria from the neurodevelopmental disorders outpatient clinic of Goztepe Prof. Dr. Suleyman Yalcin City Hospital. The diagnosis of ASD was established by an experienced psychiatrist based on Diagnostic and Statistical Manual of Mental Disorders, Fifth Edition (DSM-5) criteria through a comprehensive clinical assessment.

Inclusion criteria were as follows: being between 18 and 25 years of age, having a diagnosis of Autism Spectrum Disorder (ASD) based on DSM-5 (Diagnostic and Statistical Manual of Mental Disorders Fifth Edition, 2013) criteria through comprehensive clinical evaluation, being willing to participate in the study, and having an overall IQ score of 70 or above. Intellectual functioning was assessed using standardized Wechsler intelligence scales. Existing WISC-R scores were used when considered representative of current intellectual functioning; otherwise, the WAIS-R was administered. Participants were excluded if they had intellectual disability (IQ < 70); presence of a severe psychiatric disorder (such as schizophrenia spectrum and other psychotic disorders, bipolar and related disorders, or severe major depressive disorder); current alcohol or substance use disorder; major neurological disorders (e.g., stroke, epilepsy, brain tumor, traumatic brain injury, or central nervous system infection); chronic infectious diseases (e.g., HIV infection, hepatitis B or C, or active tuberculosis); autoimmune or chronic inflammatory diseases; active malignancy; acute infectious illness at the time of blood sampling; pregnancy, breastfeeding, or the postpartum period; or receiving systemic corticosteroid or immunosuppressive treatment.

Healthy controls were recruited from hospital staff and the local community. Controls had no current or lifetime psychiatric disorder, as determined by clinical interview.

The study protocol was approved by the Institutional Ethics Committee of Istanbul Medeniyet University (Approval No: 2026/06-08, Date: 3 June 2026). Written informed consent was acquired from all subjects before enrolment. All study procedures were performed in accordance with the ethical principles of the Declaration of Helsinki.

### 2.2. Measures

Socio-demographic Information Form. Sociodemographic and clinical data, including age, sex, BMI, smoking status, and the presence of medical comorbidities, were recorded using a data collection form developed by the researchers.

Autism Spectrum Quotient. This is a self-report questionnaire developed to quantify autistic characteristics in adults [21]. It consists of 50 items rated on a four-point Likert scale. Total scores range from 0 to 50, with higher scores indicating more pronounced autistic traits. Its original reliability coefficient was 0.64. In the Turkish adaptation, the validity and reliability coefficients were 0.72 [22].

### 2.3. Blood Sample Collection

Venous blood samples were collected from all participants between 08:30 and 11:00 a.m. following an overnight fast. Blood samples were taken in EDTA-containing tubes and centrifuged at 3000 rpm for 15 min at 4 °C. The isolated plasma samples were aliquoted and kept at −80 °C until the analysis. suPAR and CRP concentrations were determined using commercially available enzyme-linked immunosorbent assay (ELISA) kits (Elabscience Biotechnology Inc., Wuhan, China) according to the manufacturers’ instructions. All laboratory analyses were performed by investigators who were blinded to the participants’ group allocation.

### 2.4. Statistical Analyses

Statistical analyses were performed using IBM SPSS Statistics version 30 (IBM Corp., Armonk, NY, USA). Continuous variables were initially assessed for normality by visual inspection of histograms and Q–Q plots together with the Shapiro–Wilk test. Normally distributed variables were expressed as mean ± standard deviation (SD), whereas non-normally distributed variables were summarized as median and interquartile range (IQR). Categorical variables were presented as frequencies and percentages.

Group differences in continuous variables were evaluated using the independent-samples *t*-test or the Mann–Whitney *U* test, as appropriate. Categorical variables were compared using Pearson’s χ^2^ or Fisher’s exact test.

Because CRP and suPAR concentrations showed a skewed distribution, natural logarithmic transformation was applied before further analyses to better satisfy the assumptions. Hierarchical multiple linear regression analyses were performed separately for log-transformed CRP and log-transformed suPAR concentrations. In both models, diagnostic group (ASD vs. healthy controls) was entered in the first block. Age and sex were added in the second block, followed by BMI and smoking status in the final block. Variables included in the multivariable models were chosen a priori based on their established associations with peripheral inflammatory biomarkers. For the primary hierarchical multiple regression analysis, a power calculation based on a fixed-model R^2^ increase framework indicated that a minimum sample size of 81 participants would be required to detect a small-to-moderate effect of diagnostic group (Cohen’s f^2^ = 0.10) with 80% power at a two-sided α level of 0.05, assuming diagnostic group as the single tested predictor and five predictors in the full model.

Regression assumptions were tested using standardised residuals, normal probability (Q–Q) plots, homoscedasticity of residuals, multicollinearity using variance inflation factors (VIF), and influential observations using Cook’s distance.

Spearman’s rank correlation coefficient was used to examine the relationships between inflammatory biomarkers and autistic traits. Correlation analyses were performed between log-transformed CRP and suPAR concentrations and total Autism Quotient (AQ) scores.

Since plasma CRP concentrations > 10 mg/L are generally considered to indicate acute inflammatory or infectious conditions rather than chronic low-grade inflammation, an additional sensitivity analysis was performed excluding participants with CRP concentrations > 10 mg/L. The fully adjusted regression models were repeated in this restricted sample to evaluate the robustness of the primary findings.

All statistical tests were two-tailed, and a *p* value < 0.05 was considered statistically significant.

## 3. Results

### 3.1. Participant Characteristics

A total of 75 participants were included in the study, comprising 38 adults with high-functioning autism spectrum disorder (ASD) and 37 healthy controls. The sociodemographic and clinical characteristics of the participants are summarized in Table 1.

Participants with ASD were significantly younger than healthy controls (22.2 ± 2.2 vs. 24.2 ± 3.6 years, *p* = 0.007). They also had a significantly higher body mass index (BMI) (25.9 ± 4.6 vs. 23.0 ± 3.7 kg/m^2^, *p* = 0.004). The proportion of female participants was higher in the controls (45.9% vs. 23.7%, *p* = 0.043), whereas current smoking was more common among healthy controls (32.4% vs. 10.5%, *p* = 0.021). The prevalence of medical comorbidities did not differ significantly between the groups (*p* = 0.430).

### 3.2. Comparison of Inflammatory Biomarkers Between Groups

Plasma CRP and suPAR concentrations were compared between participants with high-functioning ASD and healthy controls (Table 2). Participants with ASD showed significantly higher plasma CRP concentrations than healthy controls (median [IQR]: 1.11 [1.56] vs. 0.60 [0.65] mg/L; *U* = 443.0, *p* = 0.006). In contrast, plasma suPAR concentrations did not differ significantly between the two groups (median [IQR]: 1.38 [2.33] vs. 0.96 [0.98] ng/mL; *U* = 571.5, *p* = 0.163).

### 3.3. Multivariable Analyses of suPAR and CRP Concentrations

To examine whether ASD diagnosis was independently associated with CRP and suPAR concentrations after adjustment for potential confounding variables, hierarchical multiple linear regression analyses were performed using log-transformed CRP and suPAR concentrations as the dependent variables (Table 3).

For CRP, ASD diagnosis was significantly associated with higher CRP concentrations in the unadjusted model (B = 0.687, β = 0.277, *p* = 0.016). This association remained significant after adjustment for age and sex (B = 0.857, β = 0.346, *p* = 0.006). However, after additional adjustment for BMI and current smoking status, the association between ASD diagnosis and CRP concentrations was attenuated and no longer statistically significant (*B* = 0.404, β = 0.163, *p* = 0.181). In the fully adjusted model, BMI emerged as the only independent predictor of CRP concentrations (*B* = 0.145, β = 0.511, *p* < 0.001).

For suPAR, no significant association was observed between ASD diagnosis and suPAR concentrations in either the unadjusted or adjusted models. In the fully adjusted model, the association remained nonsignificant (*B* = 0.115, β = 0.072, *p* = 0.612). Furthermore, none of the covariates, including age, sex, BMI, and current smoking status, were independently associated with suPAR concentrations in the fully adjusted model (Table 3).

### 3.4. Correlations Between Inflammatory Markers and Autistic Traits

The associations between inflammatory markers and autistic traits were evaluated using Spearman’s rank correlation analysis (Table 4). Log-transformed CRP concentrations showed a statistically significant positive correlation with AQ total scores in the overall sample (ρ = 0.261, *p* = 0.026). In contrast, no significant association was observed between log-transformed suPAR concentrations and AQ total scores (ρ = 0.073, *p* = 0.539).

### 3.5. Sensitivity Analyses

To examine whether the findings were influenced by participants with evidence of possible acute inflammation, sensitivity analyses were performed after excluding individuals with plasma CRP concentrations > 10 mg/L.

The results were largely consistent with those of the primary analyses. Eight participants (3 ASD, 5 controls) with CRP concentrations > 10 mg/L were excluded. Following exclusion of participants with CRP concentrations > 10 mg/L, plasma CRP concentrations remained significantly higher in the ASD group (*p* = 0.006), whereas no between-group difference was observed for suPAR concentrations (*p* = 0.141). In the adjusted regression model, ASD diagnosis remained unrelated to plasma suPAR concentrations. For CRP, the association between ASD diagnosis and CRP concentrations was attenuated but remained close to statistical significance (*B* = 0.452, β = 0.275, *p* = 0.055), after adjusting for age, sex, BMI and smoking status. BMI was no longer independently associated with CRP concentrations in this restricted sample.

## 4. Discussion

To the best of our knowledge, this is the first study to investigate circulating suPAR concentrations in ASD while concurrently evaluating CRP as a complementary inflammatory marker. Three principal findings emerged from the present study. First, individuals with high-functioning ASD exhibited higher circulating CRP concentrations than healthy controls in the unadjusted analyses; however, this association was no longer significant after adjustment for BMI and smoking status. Second, circulating suPAR concentrations did not differ between the ASD and control groups in either the unadjusted or adjusted analyses. Third, although CRP showed a weak positive correlation with Autism Spectrum Quotient (AQ) total scores across the total sample, no such association was observed for suPAR.

Accumulating evidence suggests that immune dysregulation may be involved in the pathophysiology of ASD, yet findings regarding peripheral inflammatory biomarkers remain inconsistent. Recent systematic reviews and meta-analyses have demonstrated increased circulating concentrations of several pro-inflammatory cytokines, including IL-1β, IL-6, TNF-α, and IFN-γ, together with reduced levels of anti-inflammatory mediators such as IL-10 and IL-1 receptor antagonist, suggesting altered inflammatory signaling in ASD [23,24,25]. Potential links between immune dysregulation and ASD may involve interactions with neurodevelopmental processes, including synaptic development and neuronal connectivity, although the nature and clinical significance of these relationships remain uncertain [6]. Nevertheless, individual studies have yielded conflicting findings, with some reporting no significant differences in inflammatory markers [26,27]. These discrepancies are likely multifactorial and may reflect differences in age, clinical phenotype, medical comorbidities, and methodological approaches across studies. Indeed, increasing evidence suggests that inflammatory alterations may differ across the autism spectrum, with distinct cytokine profiles reported in different ASD phenotypes [28,29]. Moreover, inflammatory biomarkers in adults with high-functioning ASD remain poorly characterised, and only a few studies have specifically investigated this clinically distinct subgroup, while most of the available studies were performed in children and adolescents or clinically heterogeneous ASD cohorts [14,16].

The present study showed significantly higher CRP concentrations in adults with high-functioning ASD in the unadjusted analyses. This association remained significant after adjustment for age and sex but was attenuated and no longer statistically significant following additional adjustment for BMI and smoking status, with BMI emerging as the strongest independent predictor of CRP concentrations. These findings suggest that the observed elevation in CRP may be partly explained by adiposity and other lifestyle-related factors rather than being independently associated with ASD diagnosis. The influence of adiposity on CRP is biologically plausible, as adipose tissue is an important source of pro-inflammatory cytokines, particularly interleukin-6, which stimulates hepatic CRP production [30]. Obesity is therefore increasingly recognized as a state of chronic low-grade systemic inflammation [31]. Smoking may also contribute to a persistent pro-inflammatory state through mechanisms involving oxidative stress, endothelial dysfunction, and activation of inflammatory pathways, and has been associated with alterations in a range of circulating inflammatory markers [32,33]. Although elevated CRP concentrations have been reported in ASD populations [34], to our knowledge, this is the first study to specifically evaluate circulating CRP concentrations in adults with high-functioning ASD. Thus, our results add to the existing literature by showing that the apparent association between CRP and high-functioning ASD is substantially attenuated when metabolic confounders are considered. These findings emphasise the importance of considering metabolic factors when interpreting peripheral inflammatory biomarkers in ASD, and may at least in part explain the inconsistent findings reported in previous studies.

suPAR has recently attracted increasing attention as a relatively stable indicator of persistent systemic immune activation and has been implicated in several psychiatric disorders, including schizophrenia and mood disorders. Unlike CRP, which primarily reflects current systemic inflammatory activity and is sensitive to acute inflammatory stimuli, suPAR is considered a more stable indicator of persistent immune activation and is less susceptible to short-term physiological fluctuations [17]. However, we found no evidence for elevated circulating suPAR concentrations in adults with high-functioning ASD, despite these biological characteristics. While these results do not support the existence of generalised chronic systemic immune activation as reflected by suPAR, they should not be taken as evidence against the involvement of immune mechanisms in ASD. Rather, it may indicate that the immune alterations associated with high-functioning ASD are more subtle, involve biological pathways not adequately captured by suPAR, or are influenced by co-occurring conditions and lifestyle-related factors. In addition, the available literature suggests that inflammatory alterations in high-functioning ASD may differ according to sex. Notably, two of the three available studies in this patient group either exclusively included males or identified inflammatory alterations only among male participants [14,15,16]. Given that our study included both males and females and the sex distribution differed between groups, it is also possible that sex-related biological differences contributed to the absence of detectable group differences in circulating suPAR concentrations. Although sex was included as a covariate in the adjusted analyses, larger studies with sex-balanced samples are needed to determine whether inflammatory biomarker profiles differ between males and females with high-functioning ASD.

The overall pattern of findings was largely consistent in the sensitivity analyses excluding participants with CRP concentrations > 10 mg/L. Although the association between ASD diagnosis and CRP approached statistical significance in the adjusted model, no significant association was observed for suPAR. These findings suggest that the inclusion of participants with higher CRP concentrations did not substantially alter the overall pattern of results.

We also observed a positive correlation between CRP concentrations and AQ total scores across the overall sample, whereas no association was found for suPAR. Previous studies examining the relationship between peripheral inflammatory markers and autistic traits have yielded mixed findings. Several studies in ASD samples have reported associations between higher levels of CRP or pro-inflammatory cytokines and greater symptom severity, including impairments in social functioning and stereotyped behaviours [35,36,37,38,39]. However, these associations have not been consistently replicated, with other studies finding no significant relationship between inflammatory markers and symptom severity [40,41]. The absence of a corresponding association for suPAR may indicate that different peripheral inflammatory biomarkers capture distinct aspects of inflammatory activity. Given the modest magnitude of the observed association, however, this finding should be interpreted cautiously. Further studies using dimensional measures of autistic traits across both clinical and non-clinical populations may help clarify the relationship between peripheral inflammation and ASD symptoms.

Several strengths of the present study should be acknowledged. First, to the best of our knowledge, this is the first study to evaluate circulating suPAR concentrations in ASD. Second, by focusing on adults with high-functioning ASD, we attempted to reduce the clinical heterogeneity that has limited the interpretation of previous biomarker studies. Moreover, inflammatory alterations in this clinically important subgroup remain poorly characterised, with only a limited number of studies addressing this population to date. Third, inflammatory biomarkers were evaluated using multivariable regression analyses adjusting for important demographic and metabolic confounders, and the robustness of the findings was further supported by sensitivity analyses excluding participants with evidence of possible acute inflammation.

The findings should also be interpreted in light of several limitations. First, the final sample size (*n* = 75) was slightly below the minimum sample size of 81 estimated for the primary multivariable analysis, which may have reduced the statistical power to detect small-to-moderate associations, particularly for suPAR. Therefore, the nonsignificant findings for suPAR should be interpreted cautiously and should not be considered evidence of equivalence or absence of an association. Replication in larger samples is warranted. In addition, the ASD and control groups differed in several baseline characteristics, including age, sex, BMI, and smoking status. Although these variables were included as covariates in the multivariable analyses, statistical adjustment may not fully account for these baseline differences. Second, the cross-sectional design precludes conclusions regarding causality or temporal relationships between inflammatory biomarkers and ASD. Third, only two peripheral inflammatory biomarkers were assessed; therefore, the present findings cannot exclude alterations in other inflammatory pathways or central immune processes. Finally, although major medical conditions were excluded and important confounders were adjusted for statistically, residual confounding arising from unmeasured lifestyle, environmental, or metabolic factors cannot be completely ruled out. Future studies with larger and more closely matched cohorts are needed to confirm the present findings.

## 5. Conclusions

In conclusion, adults with high-functioning ASD did not exhibit elevated circulating suPAR concentrations despite higher unadjusted CRP concentrations compared with healthy controls. The attenuation of CRP group differences after adjustment for BMI and smoking highlights the importance of considering metabolic and lifestyle factors in this population. The absence of elevated suPAR concentrations suggests that alterations in circulating suPAR may not be a consistent feature of high-functioning ASD in adulthood. However, these findings do not exclude alterations in other inflammatory pathways. Larger longitudinal studies incorporating broader panels of inflammatory biomarkers are needed to further characterise immune alterations across ASD phenotypes.

## Figures and Tables

**Table 1:** Sociodemographic and clinical characteristics of the study participants.

	ASD (*n* = 38)	Controls (*n* = 37)	*p*
Age, years	22.2 ± 2.2	24.2 ± 3.6	0.007 ^1^
Female sex, *n* (%)	9 (23.7)	17 (45.9)	0.043 ^2^
BMI, kg/m^2^	25.9 ± 4.6	23.0 ± 3.7	0.004 ^1^
Current smoker, *n* (%)	4 (10.5)	12 (32.4)	0.021 ^2^
Medical comorbidity, *n* (%)	2 (5.3)	4 (10.8)	0.430 ^3^

ASD = autism spectrum disorder; BMI = body mass index. ^1^ Independent-samples *t* test. ^2^ Pearson χ^2^ test. ^3^ Fisher’s exact test. For continuous variables, the mean and standard deviation are shown, and for categorical variables, the numbers (percentages) are used.

**Table 2:** Comparison of inflammatory biomarkers between ASD and control groups (*n* = 75).

Biomarker	ASD (*n* = 38)	Controls (*n* = 37)	U	Z	*p*
CRP, mg/L	1.11 (1.56)	0.60 (0.65)	443.0	−2.755	**0.006**
suPAR, ng/mL	1.38 (2.33)	0.96 (0.98)	571.5	−1.394	0.163

Data are presented as median (interquartile range). ASD = autism spectrum disorder; CRP = C-reactive protein; suPAR = soluble urokinase plasminogen activator receptor. U = Mann–Whitney U statistic; Z = standardized test statistic; *p* = *p*-value. Significant group differences (*p* < 0.05) are shown in bold.

**Table 3:** Hierarchical linear regression on inflammatory markers (*n* = 75).

Predictor	Model 1		Model 2		Model 3		
	B	*p*	B	*p*	B	95% CI	*p*
**A. Outcome: CRP**							
ASD (ref. controls)	**0.687**	**0.016**	**0.857**	**0.006**	0.404	−0.193 to 1.000	0.181
Age	—	—	0.057	0.233	−0.006	−0.094 to 0.082	0.891
Female sex	—	—	−0.267	0.379	−0.358	−0.890 to 0.175	0.184
BMI	—	—	—	—	**0.145**	**0.082 to 0.208**	**<0.001**
Current smoking	—	—	—	—	−0.319	−0.941 to 0.303	0.310
*R* ^2^	0.077		0.103		0.321		
Adjusted *R*^2^	0.064		0.066		0.271		
**B. Outcome: suPAR**							
ASD (ref. controls)	0.255	0.174	0.237	0.244	0.115	−0.336 to 0.567	0.612
Age	—	—	−0.034	0.280	−0.046	−0.113 to 0.020	0.169
Female sex	—	—	−0.217	0.284	−0.228	−0.632 to 0.175	0.262
BMI	—	—	—	—	0.027	−0.021 to 0.074	0.272
Current smoking	—	—	—	—	0.108	−0.363 to 0.579	0.650
*R* ^2^	0.025		0.058		0.078		
Adjusted *R*^2^	0.012		0.018		0.011		

B = unstandardized regression coefficient; CI = confidence interval; BMI = body mass index; CRP = C-reactive protein; suPAR = soluble urokinase plasminogen activator receptor. Model 1: unadjusted; Model 2: adjusted for age and sex; Model 3: additionally adjusted for BMI and current smoking status. CRP and suPAR concentrations were natural log-transformed before analysis. Significant associations (*p* < 0.05) are shown in bold.

**Table 4:** Correlations between inflammatory markers and AQ total score (*n* = 75).

	Spearman’s ρ	*p*
CRP	**0.261**	**0.026**
suPAR	0.073	0.539

AQ = Autism Spectrum Quotient; CRP = C-reactive protein; suPAR = soluble urokinase plasminogen activator receptor. CRP and suPAR concentrations were natural log-transformed before analysis. Significant associations (*p* < 0.05) are shown in bold.

## Data Availability

The data presented in this study are available on request from the corresponding author due to ethical reasons.
